# A Usability Study of a Serious Game in Cognitive Rehabilitation: A Compensatory Navigation Training in Acquired Brain Injury Patients

**DOI:** 10.3389/fpsyg.2018.00846

**Published:** 2018-06-05

**Authors:** Milan N. A. van der Kuil, Johanna M. A. Visser-Meily, Andrea W. M. Evers, Ineke J. M. van der Ham

**Affiliations:** ^1^Department of Health, Medical and Neuropsychology, Leiden University, Leiden, Netherlands; ^2^Center of Excellence in Rehabilitation Medicine, Brain Center Rudolf Magnus, University Medical Center Utrecht and De Hoogstraat Rehabilitation, Utrecht, Netherlands; ^3^Department of Rehabilitation, Physical Therapy Science & Sports, Brain Center Rudolf Magnus, University Medical Center Utrecht, Utrecht, Netherlands

**Keywords:** spatial navigation, acquired brain injury, usability, serious game, rehabilitation, cognitive training

## Abstract

Acquired brain injury patients often report navigation impairments. A cognitive rehabilitation therapy has been designed in the form of a serious game. The aim of the serious game is to aid patients in the development of compensatory navigation strategies by providing exercises in 3D virtual environments on their home computers. The objective of this study was to assess the usability of three critical gaming attributes: movement control in 3D virtual environments, instruction modality and feedback timing. Thirty acquired brain injury patients performed three tasks in which objective measures of usability were obtained. Mouse controlled movement was compared to keyboard controlled movement in a navigation task. Text-based instructions were compared to video-based instructions in a knowledge acquisition task. The effect of feedback timing on performance and motivation was examined in a navigation training game. Subjective usability ratings of all design options were assessed using questionnaires. Results showed that mouse controlled interaction in 3D environments is more effective than keyboard controlled interaction. Patients clearly preferred video-based instructions over text-based instructions, even though video-based instructions were not more effective in context of knowledge acquisition and comprehension. No effect of feedback timing was found on performance and motivation in games designed to train navigation abilities. Overall appreciation of the serious game was positive. The results provide valuable insights in the design choices that facilitate the transfer of skills from serious games to real-life situations.

## Introduction

Serious games are games that are designed for a primary purpose other than entertainment (Michael and Chen, 2005). The key concept of serious gaming is the implementation of game attributes and game mechanisms to engage users toward achieving real-life goals. While many of these game attributes and mechanics are adapted from the entertainment video games, their underlying concepts correspond well to ideas originating in fields such as behaviorism, constructivism, and neuroscience (Yusoff et al., 2009). As such, effective implementation of goals, feedback, rules, challenges and fantasy elements enhances the motivation and engagement of users toward achieving learning outcomes (Garris et al., 2002; Yusoff et al., 2009; Charsky, 2010).

Over the past decade, serious gaming has proliferated into different areas such as healthcare, military, corporate, education and government (Susi et al., 2007). A notable application of serious gaming is its introduction into the field of neuropsychological rehabilitation. Acquired brain injuries (e.g., stroke, traumatic brain injury and brain tumors) are highly prevalent in modern society (Ma et al., 2014; Peeters et al., 2015). Cognitive and behavioral deficits resulting from acquired brain injury have a profound effect on many daily life activities of these patients (Fann et al., 1995). The aim of neuropsychological rehabilitation is to aid brain injured patients in overcoming impairments and disabilities and to facilitate a return to usual self-care and daily activities (Dobkin and Dorsch, 2013). Rehabilitation programs often span over several months and require patients to engage in repeated exercises or mental rehearsals. Furthermore, patients are often required to continue with home-based therapies after they are discharged from hospital care (Trialists, 2004). The combination of home-training, repetition of exercises, and high treatment costs provide interesting opportunities for innovative approaches such as serious gaming in rehabilitation.

A distinction can be made between physical and cognitive rehabilitation. Physical rehabilitation focusses on motor abilities and sensorimotor functioning. Serious games have been developed to aid in the rehabilitation of balance impairments (Betker et al., 2006), motor functions of the hand (Afyouni et al., 2017) and the upper limbs (Broeren et al., 2008; Yoo et al., 2014), for instance. Motor rehabilitation games take a restitution-based rehabilitation approach, in which the aim is to restore impaired functions through intense and repeated stimulation of that function (Wolf et al., 2002). Consequently, the application of serious games in physical rehabilitation benefits from the motivational and engaging components of video games. Furthermore, adaptive difficulty systems implemented through game mechanics, allow for the presentation of adequate challenges, further tailoring to the need of patients in the program.

Serious gaming in cognitive rehabilitation is less common. As of now, several serious games in cognitive rehabilitation have been developed with the intention of directly training cognitive functions by incorporating mental exercises in games (‘brain training’). Brain training games such as “Lumosity” aim to strengthen attention, working memory and executive functions (Sternberg et al., 2013). The approach taken in these programs is similar to the restitution-based rehabilitation approach taken in serious games for motor rehabilitation, as patients repeatedly perform short task with increasing difficulty. Most brain training games have been developed for healthy elderly and persons with mild cognitive impairments. Randomized controlled trial studies have been performed to assess the effectiveness of brain training games in patients with cognitive impairments as a result of brain injuries. Evidence for the effectiveness of these brain training games in this population is inconclusive, as the effects of the training generally do not generalize beyond the training itself (Zickefoose et al., 2013; van de Ven et al., 2017).

Contrary to restitution-based rehabilitation, compensation-based rehabilitation has not been thoroughly explored with serious games. Compensation training is based on the concept that cognitive deficits can be overcome by substituting different latent skills or by acquiring new skills (Dixon and Bäckman, 1999). Compensatory training is one of the most important techniques in neurologic rehabilitation of acquired brain injury (Cicerone et al., 2000, 2005, 2011). Accordingly, the Cognitive Rehabilitation Task Force of the American Congress of Rehabilitation Medicine Brain Injury Interdisciplinary Special Interest Group has recommended compensation training as standard practice for memory impairments after traumatic brain injury and stroke (Cicerone et al., 2011).

Serious games designed to train compensation strategies will have additional design considerations compared to games designed to stimulate engagement. Aside from the affective components, emphasis is placed on the cognitive and educational components of the applications. Compensation strategies trained in serious games need to be transferred to daily activities. This requires patients to have a general understanding of the cognitive function that will be compensated and their own impairments regarding this function. Novel strategies will need to be introduced and trained. Finally, patients need to learn how and when a novel strategy can be applied in real-life situations (Geusgens et al., 2007).

In the current project, we have developed a serious game for the rehabilitation of spatial navigation impairments after acquired brain injury. Navigation impairments are common among stroke patients and have profound effects on the quality of life, as patients experience reduced mobility, autonomy and spatial anxiety (van der Ham et al., 2013). Even though navigational impairments in stroke patients are prevalent, no standardized rehabilitation training is currently available. A recent article advocates a compensatory approach to the rehabilitation of navigation impaired patients (Claessen et al., 2016). Instead of focusing on the rehabilitation of impaired cognitive function (such as memory or attention), The authors propose that the rehabilitation training should focus on training patients to use an alternative navigation strategy. Claessen et al. (2016) identified patients’ impaired components of the navigational ability through an extensive diagnosis procedure in a simulated virtual environment. Based on a profile resulting from this diagnosis, patients were trained to adopt a more advantageous navigation strategy in a series of virtual reality therapy sessions provided by a neuropsychologist. The results of the navigation compensation training were promising, as patients reported that they successfully adopted novel navigation strategies in real-life situations and improved on the trained navigation abilities.

As an extension to this therapy, we have developed a serious game that trains compensatory strategy use by providing multiple navigation exercises in combination with psycho-education. The goal of this serious game is to change patients’ navigation strategy in order to improve their navigation ability in daily life. The key concepts of the virtual reality therapy are adapted into a serious game that can be used at home, without supervision of a therapist. In order to ensure the usability of the application by the target patient population, an extensive user interaction test was conducted. In this usability study, three core principles of the application were examined: interaction in 3D environments, instruction modality and feedback timing.

The game’s training components take place in open, 3D environments, which patients view and interact with from a first-person perspective. In order to promote presence and stimulate the transfer of skills trained in the game, unrestricted, realistic movement in 3D environments is required. Effective movement within the 3D environments requires intuitive and accessible human–computer interaction. The manner in which users use buttons and sensors of input devices to control software events is referred to as a control scheme. Effective control schemes are believed to have a positive effect on game performance and the affective components of a game such as enjoyment, frustration and feelings of competence (Limperos et al., 2011; McEwan et al., 2012; Rogers et al., 2015; Shin and Chung, 2017). Furthermore, input modality can affect working memory, presence and experienced realism during gameplay (Kent et al., 2012; Shafer et al., 2014; Shin and Chung, 2017). In terms of compensatory strategy training, suboptimal movement control might frustrate patients, reduce engagement, and shift attention away from the educative goals of the exercises. The first aim of current experiment was to assess the subjective experience and objective performance of movement in 3D environments using two simple movement control schemes.

The navigation training application consists of different training games. In each of the games a specific spatial skill is trained. In order for patients to integrate these skills into a compensatory strategy, patients require knowledge about the concepts that underlie the training. The concepts used in spatial cognition (e.g., egocentric navigation, mental mapping, landmark knowledge, etc.) can be particularly hard to grasp for the average user. Therefore, it was important that instructions and background information about the training concepts were presented in a format that was easy to understand for patients. As the games were presented on a multimedia computer, we had the option of presenting information using text-based or video-based instructions. Video-based instructions have the advantage of conveying graphical information supporting a narrative verbal instruction, which can be particularly useful for illustrating concepts in spatial cognition. However, the stream of information from video’s might exceed the processing capacity of viewers and have a adverse effect on comprehension and knowledge organization (Mayer and Moreno, 2003; Chiu et al., 2016). This might be of importance as working memory is particularly vulnerable for impairment after acquired brain injury (McDowell et al., 1997; Christodoulou et al., 2001). Consequently, we expected that the self-pacing nature of text-based information would allow for a more optimal transfer of knowledge in acquired brain injury patients. The second aim of the study was to determine whether text-based instructions are more effective than video-based instructions by assessing objective performance and subjective preferences in an instruction comprehension task.

Feedback presentation is an important component of effective serious game design (Garris et al., 2002; Yusoff et al., 2009; Charsky, 2010). The type, amount and timing of feedback has been shown to be of influence on learning efficacy and motivation in computer-based learning (Erhel and Jamet, 2013). The effect of feedback timing is often studied in the context of knowledge and skill assessments, where feedback is given directly after an answer is given or after a delayed period of time. Advantages and disadvantages of feedback timing on learning efficiency have been identified. Direct feedback allows learners to instantly correct erroneous responses, contributing to knowledge acquisition (Kulik and Kulik, 1988). However, processing direct feedback competes with cognitive resources required for learning process and can disrupt the learning process (Schooler and Anderson, 2008). Inversely, delayed feedback has been shown to facilitate knowledge retention over longer periods of time, but performance during knowledge acquisition is reduced (Shute, 2008). Feedback timing effects have predominantly been studied in educational scenario’s such as classroom settings, quizzes and programming courses. In these scenarios responses can be directly evaluated and responses are often clearly correct or false. Less is known about the effects of feedback timing in games where skills are taught through interaction with a virtual game world. Responses are seldom binary in games, but rather expressed in a variable such as a score. Therefore, scoreboards are often implemented to allow users to monitor their performance during the gameplay. The timing and prevalence of this scoreboard can be controlled.

The current study focused on two methods of feedback timing: cumulative feedback and delayed feedback. Cumulative feedback refers to the explicit presentation of a patient’s overall performance during gameplay. Cumulative feedback is shown directly after completing each challenge on an interval basis. Delayed feedback refers to explicit presentation of a patient’s overall performance after gameplay. The third aim of the study was to determine whether feedback timing affects objective performance and motivation (engagement and self-efficacy) during a navigation strategy training game. Cumulative feedback has been shown to positively affect performance in a working memory task compared to a no feedback condition (Adam and Vogel, 2016). Furthermore, cumulative feedback is similar to direct feedback described in more traditional feedback timing studies in the sense that patients can adjust their behavior during tasks. We hypothesized that cumulative feedback leads to increased performance during gameplay compared to delayed feedback.

The serious game will serve as a home-based rehabilitation treatment which patients will use over an extended period of time without supervision. In this usability study, three core principles of the application were examined: interaction in 3D environments, instruction modality, and feedback timing. As the game required patients to interact with 3D virtual environments, we have determined what type of movement control was most intuitive: mouse controlled movement or keyboard controlled movement. In order for the training to be effective, an understanding of complex spatial concepts was required. We therefore determined what instruction modality was most effective for the acquisition of knowledge in acquired brain injury patients: video-based instructions or text-based instructions. Furthermore, we have determined how performance and perceived competence were affected by cumulative and delayed feedback. Finally, as the serious game was designed to be effective for all patients with brain injuries, regardless of the nature of the brain injury, we assessed whether differences between brain injury types exist in the appreciation of the application.

## Materials and Methods

### Patients

A total of 30 acquired brain injury patients participated in the study (**Table 1**). All patients were included by occupational therapists at the Department of Rehabilitation of the University Medical Center Utrecht. Inclusion criteria were: (a) clinically diagnosed with acquired brain injury (e.g., cerebrovascular accident, traumatic brain injury, hypoxic-anoxic brain injury), (b) in the non-acute phase of brain injury, (c) between 18 and 80 years of age, (d) capable of operating a computer system using their left or right hand, (e) sufficient communication, comprehension and taxability (judged by an occupational therapist), (f) no visual impairments interfering with the tasks (e.g., blindness, neglect). All participants gave written informed consent before participating in the study. Patients did not receive monetary compensation for study participation.

**Table 1:** Characteristics of patients in study (*n* = 30).

Variable	
Gender, male N	15 (50%)
Age in years, mean (range)	47.2 (23-68)
Education^∗^, mean (*SD*)	5.4 (1.07*)*
Brain Injury Type	
- Cerebrovascular accident	16 (53.3%)
- Traumatic brain injury	9 (30%)
- Brain tumor	4 (13%)
- Brain hypoxia	1 (3.33%)
Brain injury location	
- Left	9 (30%)
- Right	11 (36.67%)
- Bilateral	3 (10%)
- Unspecified/Unknown	7 (23.33%)
Months after brain injury, mean (SD)	26.43 (52.71)

*^∗^Education scores used the Verhage scale. This is a Dutch education classification system including 7 categories (Verhage, 1964): 1, lowest; 7, highest.*

This study was exempted from ethical approval by the Medical Ethics Committee of the University Medical Center Utrecht in accordance with the Dutch WMO law. This study was performed in accordance with the Declaration of Helsinki and the ICH guidelines for good clinical practice.

### Tasks and Material

Three tasks were employed to assess different aspects of the software’s usability: movement control, instruction modality and feedback timing. Each task was comprised of an objective component, performance on the task, and a subjective component, a questionnaire with questions regarding a patient’s user experience (**Tables 2**, **3**). Furthermore, a questionnaire was used to assess the menu-interaction experience (**Table 4**). Additional questionnaires were presented at the start and end of the experimental session to measure computer experience and general appreciation, respectively (**Table 5** and Supplementary Table 1).

**Table 2:** Movement control questionnaire (*n* = 30).

Variable	Question	Mouse^∗∗^ mean (*SD*)	Keyboard^∗∗^	mean (*SD*)	*p^∗^*
Ease of use	I thought walking around in the environment was easy	4.2 (1.35)	3.33 (1.49)	**<0.01**
Improvement	Over time I felt I improved at walking around in the environment	4.3 (1.09)	3.9 (1.37)	0.14
Other software	The controls of this application were similar to other software I have used	3.33 (1.77)	2.86 (1.59)	0.11
Enjoyment	I enjoyed walking in the environment	4.24 (1.06)	3.72 (1.22)	**<0.01**
Presence	I could imagine myself walking in the environment	3.7 (1.26)	3.3 (1.44)	**<0.05**

*^∗^Significant differences are printed in bold letters. ^∗∗^Ratings on a Likert scale with 1 corresponding to “completely disagree” and 5 corresponding to “completely agree.” Standard deviations appear in parentheses next to means.*

**Table 3:** Feedback timing questionnaire (*n* = 21).

Type	Question	Cumulative^∗∗^ mean (*SD*)	Delayed^∗∗^ mean (*SD*)	*p^∗^*
Interest	I thought the task was interesting	4.33 (0.80)	4.57 (0.93)	0.17
Enjoyment	When I performed the task. I enjoyed myself.	4.38 (1.12*)*	4.57 (0.98)	0.26
Perceived difficulty	I thought the task was easy	2.90 (1.34)	2.90 (1.41)	0.96
Effort	I put a lot of effort into completing the task	3.86 (1.35)	3.48 (1.25)	0.32
Strive	I did the best I could during this task	4.62 (0.59)	4.48 (0.87)	0.41
Competence	I had the feeling I was good at the task	3.71 (1.27)	3.62 (1.36)	0.69
Accept results	I am content with my performance	3.57 (1.33)	3.52 (1.44)	0.79
Competition	I think my performance was above-average	2.67 (1.15)	3.10 (1.37)	0.11
Desire to improve	I wish I was better at the task	3.81 (1.36)	3.62 (1.32)	0.47

*^∗^Differences between responses in the delayed and cumulative feedback timing condition were compared per item using the Wilcoxon Signed Rank test. ^∗∗^Ratings on a Likert scale with 1 corresponding to “completely disagree” and 5 corresponding to “completely agree.” Standard deviations appear in parentheses next to means.*

#### Movement Control

**FIGURE 1:**
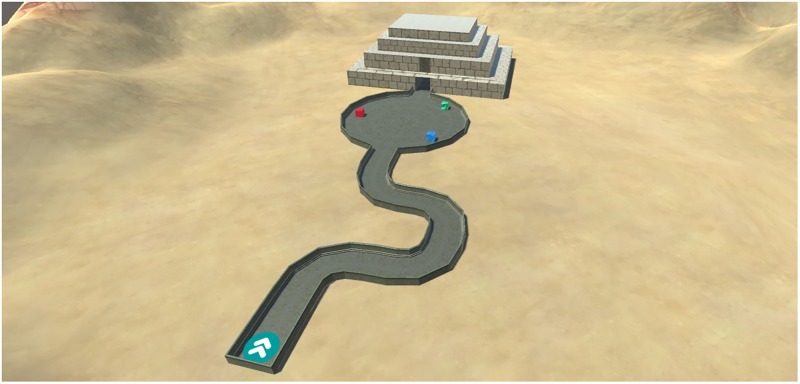
Design of the environment used in the movement control task. The environment can be subdivided in a meandering part, a circular area and a building featuring sharp turns. A mirrored version was created to accommodate for the two conditions.

**FIGURE 2:**
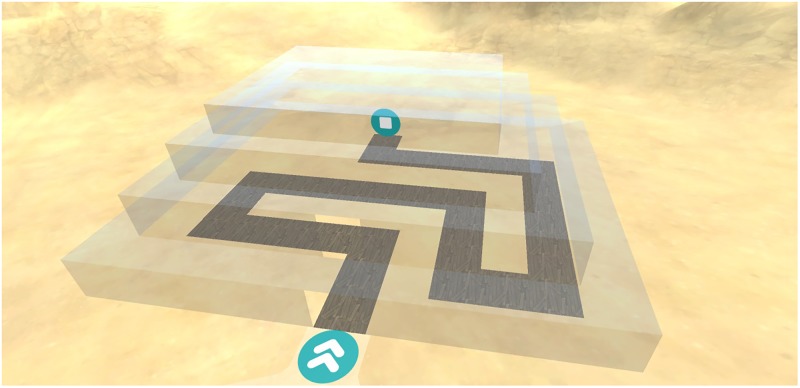
Design of the corridor with sharp turns used in the movement control task. The corridors inside the building are made up of 8, 90°; turns. The blue icon with arrows indicates the entrance of the building. The blue icon with the square indicates the end location of the task.

The movement control task was designed to assess usability differences between mouse controlled and keyboard controlled movement in 3D environments. A virtual environment was created resembling a sandy desert (**Figure 1**). A bordered plateau was placed in the middle of this environment. The plateau consisted of three distinct components: A broad meandering road, a large circular environment and a building consisting of narrow corridors and 8 90-degree turns (**Figure 2**). Three colored cubes (red, green, blue) were placed in the circular environment. The starting-location was placed at the beginning of the meandering road and the end-location was placed at the end of the corridor inside the building. Following the one-way road lead to the end-location as no junction points or crossroads were present. A geometrically mirrored version of environment was created to facilitate comparable environments for the two movement conditions.

Keyboard controlled movement was performed by pressing the four arrow keys on the keyboard. “Up” corresponded to forward movement, “down” corresponded to backward movement and the “left” and “right” buttons corresponded to left and right rotation. Mouse controlled movement was performed by using the left and right mouse button and by utilizing the optical sensor. Left mouse button corresponded to forward movement, right mouse button corresponded to backward movement, moving the mouse left or right corresponded with rotation in the respective direction. Similar to the keyboard input condition, participants were unable to look up or down using the mouse. Movement speed was set to 5 in both conditions. This corresponded to a walking velocity of approximately 5 km/hour.

**Table 4:** Menu-interaction experience (*n* = 29).

Statement	Response^∗^ mean (*SD*)
The text was easy to read	4.41 (1.09)
The information was placed where I expected it to be	4.14 (0.88)
The color and layout used in the application was distracting^∗∗^	4.62 (0.78)
The terms used in the application were comprehensible	3.93 (1.36)
I understood what was meant with the term “levels”	4.38 (1.12)
I knew what the training was about by reading the names of the games	3.89 (1.26)
It was easy to navigate between different menus	4.03 (1.27)
It was easy to view the progression that was made on different challenges	3.97 (1.35)
I thought logging in was difficult^∗∗^	4.48 (1.24)
Controlling the application was easy to learn	4.69 (0.81)
Learning what the terms meant was easy	4.14 (1.30)

*^∗^Ratings on a Likert scale with 1 corresponding to “completely disagree” and 5 corresponding to “completely agree.” Standard deviations appear in parentheses next to means. ^∗∗^Data shown on a reversed scale, higher score indicate higher ratings of usability.*

**Table 5:** Overall appreciation questionnaire (*n* = 24).

Variable	Statement	Response^∗^ mean (*SD*)
Ease of use	The software was use to use	3.63 (0.25)
Enjoyment	I enjoyed the experience	4.17 (0.23)
Clear goals	The goals were clearly defined	4.00 (0.24)
Rewarding	The experience was rewarding	3.92 (0.22)
Control	I had a feeling of total control	3.29 (0.26)
Attention	My attention was completely directed on the task at hand	4.79 (0.10)
Concentration	I was concentrated	4.54 (0.19)
Willingness to play again	I would like to play the game again	4.13 (0.23)
Challenge	The game was challenging	4.08 (0.21)

*^∗^Ratings on a Likert scale with 1 corresponding to “completely disagree” and 5 corresponding to “completely agree.” Standard deviations appear in parentheses next to means.*

Patients were placed at the start of the meandering road and were asked to travel to the end-location which was placed at the end of the corridors in the building. Before entering the building, all colored cubes had to be picked up. Cubes were picked up by bumping into them. Patients were instructed to travel to the end-location as fast as possible, without touching the walls. Time required to finish the task (seconds) and number of collisions with the walls were recorded. Patients performed a single trial in each condition. A usability questionnaire was filled in following each movement tasks. This questionnaire measured the following concepts: *ease of use*, *experienced improvement, similarity with other software*, *enjoyment* and *presence* on a 5 point Likert scale (**Table 2**). After both the mouse controlled and keyboard control tasks were completed, patients were presented with an open questionnaire consisting of four questions: (1) What method of movement did you like best? (2) Why did you prefer this method over the other? (3) Do you have suggestions on how we could further improve the movement in the game? (4) What method of movement control would like to see in the training?

#### Instruction Modality

As the serious game was designed for desktop computers, instructions could be provided using narrated video (tutorial video) as well as more traditional texts. The instruction modality task was designed to assess differences in knowledge acquisition between text-based instructions and video-based instructions. The instructions of 2 existing navigation training games were used (“sense of direction game” and the “map use game”). Text-based and video-based instructions were constructed for both games. In the video version, the text was read aloud by a narrator and supported by a video montage of a person playing the game. In the text version, text was printed on the screen and patients could scroll through the text at their own pace. When presented with the video version, patients were asked to watch and memorize the video. When presented with the text only version of the instructions, patients were instructed to read and memorize the text. No time limit was set. The order in which patients received the video-based or text-based instruction, as well as the combination of instruction modality and version of the game was counterbalanced across patients.

After observing the instructions, patients were shown 12 statements about the objectives of the game and the implications of using the navigation strategy that was trained in the game (Supplementary Tables 2, 3). Patients determined whether these statements were true or false. Following the true or false statements for both instruction modalities, participants answered three open questions: (1) What instruction type did you find most effective? (2) Why did you prefer this type of instructions? (3) Do you have suggestions on how we could further improve the instructions?

#### Feedback Timing

**FIGURE 3:**
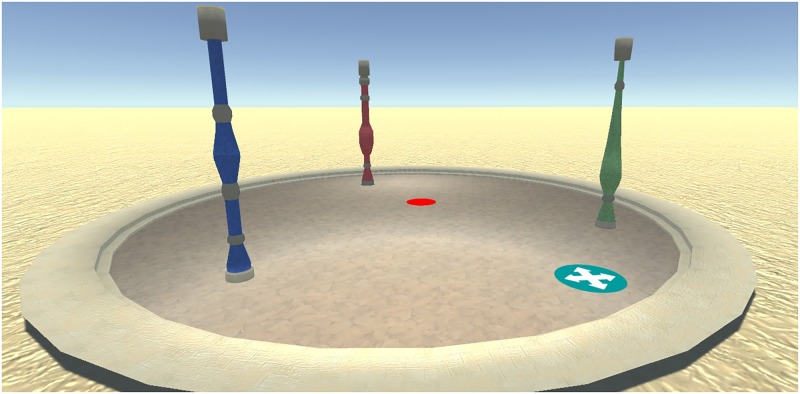
Design of the environment used in the feedback timing task. In this version of the task, participants study a map to remember the location of the goal (red dot) in relation to the landmarks (pillars). Patients were then placed on the starting location (blue dot). The goal and start locations are not visible during a round.

The feedback timing task was designed to assess the effect of cumulative vs. delayed feedback on performance and motivation during a play-through of a training game. A virtual environment was created resembling a sandy desert. In this middle of the environment, a bordered circular plateau was placed. Two versions of the game were used. In the first version, 4 distinct landmarks were placed in the north, south east and west of the plateau. These landmarks resembled the Horse of Troy, a Greek galley, a Greek temple and the Colossus. In the second version, 3 local landmarks were placed inside of the plateau. The landmarks resembled different colored pillars (red, green, blue). A hidden goal location was placed on the plateau (**Figure 3**).

At the start of a trial, a 2D map of the environment was shown on which the hidden plateau and the landmarks were highlighted. Patients were then placed in the 3D environment and were tasked to walk toward the hidden plateau, by orienting on the landmarks. The movement control was similar to the keyboard controlled movement described in the movement task above. A pedometer bar was shown at the top of the screen to indicate the amount of distance a patient had traveled. The amount of coins in possession corresponded to the size of the pedometer bar. As such, patients were instructed to take as few steps as necessary to reach the end-location. Between 0 and 2 coins could be earned in each round. The goal of the game was to earn as many coins as possible over the course of 3 rounds. In the cumulative feedback condition, a large scoreboard was presented between rounds. This scoreboard showed the percentage of coins collected over the whole trial (so if patients collected 3 coins at the end of round 2, the score would show 75%). The scoreboard allowed patients to monitor their performance of the span of 3 rounds. In the delayed feedback condition, no overall score feedback was given between rounds.

At the end of the three rounds, an overall score was shown in both conditions. The total amount of coins earned was used the measure of performance. After completing a task, patients filled in a questionnaire that measured motivational components related to engagement: *interest in task*, *enjoyment*, *effort invested while playing, strive* (I did the best I could during this task), *desire to improve*, and components related to self-efficacy: *perceived difficulty*, *competence*, *result acceptance*, *comparative score* (**Table 3**). The items were rated on a scale from 1 to 5, 1 corresponding to “completely disagree” to 5 corresponding to “completely agree.”

#### Additional Measures

The menu interaction task was designed to assess the comprehensibility of the menu structure and phrasing of terms used in the game. Patients were required complete seven tasks by navigating through the menu tabs. In each task, specific information needed to be found or specific actions were required. Patients were asked to conduct the following activities: (1) log in, (2) start a specific game, (3) locate background information about the application, (4) determine the current level on a specific game, (5) start another game, (6) determine the amount of coins (score) currently in possession, (7) quit the application. Patients were instructed to think out loud while navigating the menu screens. When patients navigated to a wrong menu or when they indicated they were unable to find the requested information, the experiment would show the correct method of finding the information. Following the menu interaction task, a usability questionnaire was filled in (**Table 4**). The questionnaire was specifically designed to address layout, comprehensibility and interaction with important items of the menu interface.

The computer experience questionnaire consisted of nine items and was rated on a 5-point Likert scale (Supplemental **Table 2**). The items in this questionnaire were inspired by the Computer Attitude Scale and the Computer User Self Efficacy scale (Nickell and Pinto, 1986; Cassidy and Eachus, 2002). The first four items of this question addressed a patient’s exposure to computers. Items 5–8 concerned a patient’s self-reported knowledge of operating software and hardware. The ninth item addressed feelings of anxiety when using a computer.

The overall appreciation questionnaire consisted of nine items and was rated on a 5-point Likert scale (**Table 5**). Six items in this questionnaire were adapted from the Flow State Scale and three items constructed in context of the usability test (Jackson and Marsh, 1996). The items addressed the overall appreciation of the application and the experience of flow during the tasks. The items were rated on a scale from 1 to 5, 1 corresponding to “completely disagree” to 5 corresponding to “completely agree.”

The tasks were constructed in the Unity 3D game engine, version 5.3.4.4.f1, and run as standalone applications. The application was run on a HP EliteBook 8760w laptop with a NVIDEA Quadro 3000M graphic processing unit. The laptop’s screen size was 17.3-inch wide screen (15.5^∗^ 8.98) inch. The laptop’s keyboard and a standard desktop mouse model (Dell Optical Mouse – MS116) were used as input devices. All questionnaires were constructed in Qualtrics and presented using an internet browser.

### Procedure

The data was collected in a therapy room of the Department of Rehabilitation of the University Medical Center Utrecht. All patients read the study’s information letter in advance and gave written informed consent prior to the session. All experimental sessions were planned prior to or after a patient’s scheduled appointment with a doctor or occupational therapist. In order to comply to a patient’s schedule during the visit to the medical center, each experimental session was brought to an end after approximately 60 min of testing.

At the start of the experimental session, patients were informed about the nature of the study. Patients were explicitly informed about the study’s objective of tailoring the software to patients’ capability and needs. As such, patients were encouraged to ask questions about the software, discuss design choices and propose suggestions for changes in the software’s design. To stimulate communication with the patients, an informal and relaxed atmosphere was pursued.

The experiment started with the computer experience questionnaire. This was followed by the movement control task, the instruction modality task, the menu navigation task and finally the feedback task. Patients then filled in the overall appreciation questionnaire.

### Statistical Analyses

#### Analysis of Objective Performance

Objective performance in the movement control, instruction modality and feedback timing tasks was analyzed using within-subject tests. Data were tested for normality using Kolmogorov–Smirnov tests. Normally distributed data were analyzed using a three-way mixed model ANOVAs with (condition) as within subject factor and (brain injury type) and (brain injury location) as between subject factors. Non-normal data were analyzed using Wilcoxon signed-rank tests, in which conditions were contrasted. Separate Kruskal–Wallis *H* Tests were used to assess the effects of brain injury type and brain injury location on performance in non-normal datasets.

#### Analysis of Subjective Measures

Internal reliability analyses were performed on all questionnaires. Non-parametric tests were used to analyze the effect of condition on subjective measures. Additionally, the proportion of responses for the preference (*what condition did you prefer*?) items in the open questionnaires were analyzed using Chi-square tests of independence. The effects of brain injury type and brain injury location on subjective responses were assessed using Kruskal–Wallis tests.

#### Exploratory Analysis

Exploratory analyses were performed to inspect the relation between objective performance and subjective measures for the movement control task and the feedback timing task. Pearson correlations analyses were conducted to investigate the relation between objective performance and items of the subjective measure questionnaires.

#### Attrition

Six patients were unable to complete all tasks of the experiment within 60 min. Additionally, 2 patients were unable to complete the instruction modality task due to reading impairments. One patient was unable to complete the feedback timing task due to severe navigation impairments. Technical difficulties lead to missing data of 1 patient in the movement control task and 2 patients in the feedback timing task. As such, the sample size for the objective performance analysis for the movement task was 29 (30 for the subjective measures), the sample size of the instruction task was 27(29 for the preference response) and the sample size of the feedback timing task was 21.

## Results

### Movement Control

In order to compare objective movement performance in the mouse and keyboard controlled conditions, time required to finishing the task (time) and the number of collisions with the walls (wall bumps) were analyzed as main measures. A Kolmogorov–Smirnov test indicated that the data for time (mouse), *D*(29) = 0.21, *p* < 0.01 and wall bumps (keyboard), *D*(29) = 0.17, *p* < 0.05, were both significantly non-normal.

**FIGURE 4:**
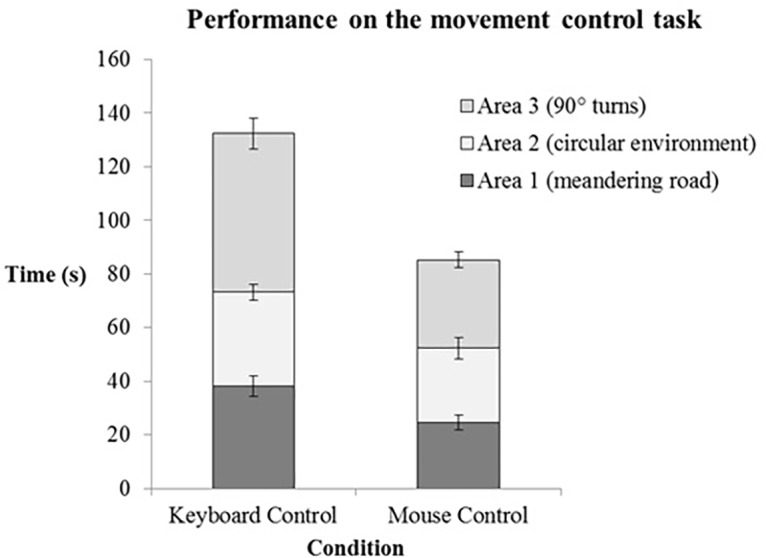
Performance on the movement task for keyboard and mouse controlled movement (*n* = 29). The average time spend (seconds) in each area is indicated by the different colored stacks in the graph. The error bars represent the standard error of the mean.

A Wilcoxon signed-rank test revealed that time in the mouse control condition (*M* = 85.29, *SD* = 44.19) was significantly shorter than time in the keyboard control condition (*M* = 132.42, *SD* = 58.63), *z* = -4.68, *p* < 0.01, *r* = -0.61 (**Figure 4**). No significant effects of condition were found on the number of wall bumps *z* = -0.92, *p* = 0.36, *r* = -0.12. Additional Wilcoxon signed-rank tests were performed to compare the effects of movement control type within in the three sections of the environment. Mouse controlled movement was faster than keyboard-controlled movement in the meandering area (*p* < 0.01) the circular area (*p* < 0.01) and the area with the sharp turns (*p* < 0.01) (**Figure 4**).

A Kruskal–Wallis *H* Test revealed that there was no effect of brain injury type on performance in the keyboard [χ^2^(3) = 3.71, *p* = 0.29] and mouse controlled [χ^2^(3) = 5.49, *p* = 0.14] movement tasks. Similarly, no effect of brain injury location was found on performance in the keyboard [χ^2^(3) = 1.99, *p* = 0.57] and mouse controlled [χ^2^(3) = 2.94, *p* = 0.40] movement task.

After completing the movement task, patients filled in a subjective preference questionnaire. A reliability analysis was performed and revealed an internal reliability of α = 0.85 for the keyboard condition and α = 0.69 for the mouse condition. Each of the 5 items of the questionnaire were compared for the mouse control and keyboard control condition using a Wilcoxon signed-rank test. A significant effect of condition was found for ease of use, as the mouse controls (*M* = 4.2, *SD* = 1.35) were rated as easier to use than keyboard controls (*M* = 3.33, *SD* = 1.49), *z* = -2.67 *p* < 0.01, *r* = -0.34. Mouse control (*M* = 4.24, *SD* = 1.06) was also rated as significantly more *enjoyable* than keyboard control (*M* = 3.72, *SD* = 1.22 ), *z* = -2.67, *p* < 0.01, *r* = -0.34. Furthermore, a higher level of *presence* was experienced during mouse controlled movement (*M* = 3.7, *SD* = 1.26) compared to the keyboard control (*M* = 3.3, *SD* = 1.44), *z* = -2.36, *p* < 0.05, *r* = -0.30 (**Table 2**).

Analysis of the open questionnaire revealed that 90.0% of the patients reported a preference for mouse controls, 10% of the patients reported a preference of keyboard control and 0% of the patients did not have a clear preference. A Chi-square test of independence revealed a significant difference in proportions, χ^2^(1) = 19.20, *p* < 0.01.

Using Spearman correlation analyses, the relation between objective performance (time) in the movement tasks and the ratings on the 5 items of the questionnaire was explored. A correlation between objective performance and *enjoyment* was found for both the mouse control, *r* = 0.43, *p* < 0.05, and keyboard control *r* = 0.39, *p* < 0.05, conditions. Additionally, a correlation between objective performance and *presence* was found for both the mouse control, *r* = 0.41, *p* < 0.05, and keyboard control *r* = 0.40, *p* < 0.05, condition.

### Instruction Modality

In order to determine the effect of instruction modality on learning, patients answered 12 true of false questions about the content of the instructions. Percentage correct was compared for the video-based and text-based condition. A Kolmogorov–Smirnov test indicated that the video-based instruction data was significantly non-normal *D*(27) = 0.19, *p* < 0.05. A Wilcoxon signed rank test was used to compare percentage correct for the video-based and text-based condition. No significant effect of instruction modality was found, *z* = -0.82, *p* = 0.41*, r* = -1.12. Percentage correct did not differ between the video-based (*M* = 70.20, *SD* = 15.64 ) and text-based (*M* = 66.13, *SD* = 17.25) condition.

A Kruskal–Wallis *H* Test revealed that there was no effect of brain injury type on percentage correct in the video-based [χ^2^(2) = 1.78, *p* = 0.41] and text-based [χ^2^(2) = 1.01, *p* = 0.60] conditions. Furthermore, no effect of brain injury location was found on the percentage correct in the video-based [χ^2^(3) = 0.9, *p* = 0.83] and text-based [χ^2^(3) = 1.09, *p* = 0.78] conditions.

The proportion of self-reported instruction preference was investigated using a chi-square test of independence. 65.51% of participants indicated a preference for the video-based instructions compared while 20.69% of the patients preferred the text-based instructions. 13.79% of the participants did not have a clear preference. The chi-square test revealed that this difference in proportions was significant, χ^2^(2) = 13.72, *p* < 0.01.

### Feedback Timing

The effect of feedback timing on objective performance was investigated by comparing the total amount of coins between the cumulative and delayed feedback condition. The total score was calculated by summing the amount of coins over three rounds for the cumulative feedback (*M* = 3.48, *SD* = 1.63) and delayed feedback (*M* = 3.95, *SD* = 1.75) tasks (Supplementary Table 4). A Kolmogorov–Smirnov test indicated that the total score (cumulative), *D*(21) = 0.15, *p* = 0.2 and total (delayed), *D*(21) = 0.17, *p* = 0.14 were normally distributed.

A three-way repeated measures ANOVA was performed to compare the effect of feedback timing on total score in the delayed and cumulative feedback condition with brain injury type and brain injury location as between subject factors. No significant main effect of condition was found *F*(1,12) = 0.13, *p* = 0.27, η_p_^2^ = 0.10. No significant interaction effect was found for brain injury type and condition (*p* = 0.41) and brain injury location (*p* = 0.73).

After completing the feedback timing task, patients filled in the motivation questionnaire. Each of the 9 items of the questionnaire were compared between the cumulative and delayed feedback conditions using a Wilcoxon signed-rank test. No significant effect of condition was found in any of the items (**Table 3**).

In an explorative analysis, the relation between objective scores on the feedback tasks and ratings on the questionnaire were analyzed using Spearman correlations. In delayed feedback condition, a significant relation was found between objective score and ratings in *perceived difficulty*, *r* = 0.59, *p* < 0.01, *competence*, *r* = 0.55, *p* < 0.01, *result acceptance*, *r* = 0.74, *p* < 0.01 and *competition*, *r* = 0.73, *p* < 0.01. The subjective raring on the items correlated in a positive linear fashion with the objective score.

Similar relations were found between objective score and self-reported ratings on the cumulative feedback condition. Objective score significantly related to *perceived difficulty*, *r* = 0.61, *p* < 0.01, *competence*, *r* = 0.64, *p* < 0.01, *result acceptance*, *r* = 0.72, *p* < 0.01 and *competition*, *r* = 0.57, *p* < 0.01. The subjective raring on the items correlated in a positive linear fashion with the objective score. Additionally, a strong negative relation was found between *desire to improve*, *r* = -0.65, *p* < 0.01, and objective performance. The rating on the desire to improve item correlated negatively with objective score in linear fashion.

### Additional Measures

After performing the menu interaction tasks, patients rated the usability of the menu navigation (**Table 4**). The 11 item questionnaire showed a high internal reliability of α = 0.81. An overall score of the menu-navigation was computed by averaging the ratings of each item. A Kruskal–Wallis test was conducted to compare appreciation ratings between brain injury type and between brain injury location. No effect of brain injury type or location was found on the ratings on the overall menu interaction questionnaire.

The overall appreciation questionnaire was filled in at the end of the session to obtain ratings of overall appreciation and the experience of flow (**Table 5**). The 9 items of this questionnaire yielded a reliability rating of α = 0.76. An overall rating of appreciation questionnaire was computed by averaging the ratings of each item. A Kruskal–Wallis test was conducted to compare usability rating between brain injury types and brain injury locations. No effect of brain injury type or location was found on the ratings on the overall appreciation of the game.

## Discussion

The usability of a serious game designed to train compensatory navigation strategies in acquired brain injury patients was investigated. The usability of three core principles of the application was examined using objective and subjective measures: movement control, instruction modality and feedback timing.

Intuitive control schemes in games contribute to motivation, engagement and reduction of cognitive load (Limperos et al., 2011; McEwan et al., 2012). The importance of responsive controls in serious games has been identified by several guidelines and frameworks concerned with usability (Pinelle et al., 2008). In order to optimize interactivity with the virtual environments used in the game, two control types were assessed: mouse and keyboard. The acquired brain injury patients clearly preferred mouse controlled movement over keyboard controlled movement. Mouse controlled movement was rated easier to use, more enjoyable and a stronger feeling of presence in the environment was experienced. While there is no consensus about the positive effects of presence in training programs, several studies have suggested that high levels of presence might aid in the transfer of skills acquired during the training (Youngblut and Huie, 2003; Alexander et al., 2005; Stevens and Kincaid, 2015). The advantages of mouse controlled movement over keyboard controlled movements were reflected in the objective performance measurements. Time required to finish the tasks was lower is using the mouse, while the number of wall collisions between control type did not differ. This indicates that patients did not lower accuracy in favor of speed when using mouse controlled input. Additionally, mouse controlled movement was faster in all three areas of the environment, revealing that the advantages of mouse movement were not specific to a single maneuver, such as taking sharp turns. An exploratory analysis revealed a positive relation between objective performance and ratings of enjoyment and presence in the environment in both movement control conditions. This finding further supports the notion that effective interaction results in a more enjoyable and natural gameplay experience. In sum, the implementation of simple, mouse controlled movement in 3D environments is recommended over keyboard-controlled movement based on objective and subjective evidence in this study.

Unrestricted movement in virtual environments allows patients to develop and experiment with novel navigation strategies. However, patients can only progress through the game when specific strategies are successfully adapted. It is therefore important that the underlying concepts of the compensatory strategies are clearly communicated. Computers are multimedia systems that allow for different instruction modalities. In the current experiment, we examined the effects of video-based and text-based instruction on knowledge acquisition. No clear learning advantages of video-based instructions over text-based instruction were found. Similar results are found in studies that assess knowledge acquisition of complex topics (the news) through printed text and video (Furnham and Gunter, 1985; Walma van der Molen and Van Der Voort, 2000). While the results do not indicate an advantage for either modality, a clear preference for the video-based instructions was found in the questionnaire responses. During conversations with the patients about their preferred instruction modality, patients mentioned the advantage of visual information in explaining spatial concepts. This discrepancy between performance and preference can be explained in terms of cognitive capacity. Patients recognized that more information was presented to them in the video condition compared to the text condition. However, this additional information was not effectively maintained. We suspect that the continuous stream of information in the instruction video might have disrupted the information encoding process. Capacity constrains were not limited to the video-based instructions. Two patients were unable to complete the text-based instruction task due to their impairments. While these patients were able to read short texts, they were incapable of maintaining their attention when reading extensive bodies of text. The overload of cognitive capacity can be managed by providing patients with additional control over the pacing of the video (Mayer and Moreno, 2003). The aim for the instructions in the current game is to provide short and effective information before starting a gaming module. In this context, requiring patients to systematically analyze a video might not be an optimal solution. Subsequently, the addition of visual static images to text-based instructions might be more effective than both video-based and solely text-based instruction. This suggestion is supported by studies with healthy subjects (Mayer et al., 2005). More research is required to determine if this combination will indeed enhance knowledge acquisition in acquired brain injury patients. Overall, in this study we have established that patients prefer video-based instructions over text-based instructions. Video-based instructions are not more effective in context of knowledge acquisition and comprehension.

Feedback presentation is an important component in education and serious gaming (Garris et al., 2002; Yusoff et al., 2009; Charsky, 2010). Contrary to our expectation, we did not find a beneficial effect of cumulative feedback on objective performance. Updating patients on their overall score between rounds did not enhance performance in the task. Furthermore, the motivational components of the game were not affected by the timing of feedback as cumulative feedback did not affect engagement and self-efficacy. An earlier study showed beneficial effects of cumulative feedback on performance in a working memory tasks when compared to a no-feedback condition (Adam and Vogel, 2016). There might be several reasons why this effect was not observed in the current study. First, the current task included only 3 trials per condition, whereas Adam and Vogel (2016) employed 150 short trials. It is possible that the beneficial effects of cumulative feedback only arise after participants are familiar with the task and start performing at a stable level. In the current task, it is possible that participants were still experimenting with strategies to complete the task. Second, the current task was considerably more complex than the working memory task employed by Adam and Vogel (2016). This might have lead to a greater variation in performance in both feedback timing conditions. Another explanation for this finding is that patients were not heavily invested in their performance within the game, as patients were explicitly informed that the goal of the study was to test the usability of the application. However, further analysis revealed positive linear relations between objective score and result acceptance (“*I am happy with my performance*”), indicating that patients were indeed concerned with their score. The exploratory analysis also revealed a negative linear relation between willingness to improve (“*I wish I was better at the task”)* and the objective score in the cumulative feedback condition. This finding hints at a subtle effect of cumulative feedback on motivation. It is, however, unclear whether this effect is beneficial or disadvantageous, as this statement can be interpreted as a lack in confidence induced by the feedback or an increase in motivation to perform better. Overall, the current experiment did not provide evidence for the advantageous learning or motivational effects of cumulative feedback over delayed feedback.

Interaction with the menu screens and the overall appreciation of the game were evaluated positively. Importantly, neither the type of brain injury nor the location of the brain injury affected ratings on the appreciation and menu interaction questionnaires. Similarly, no effect of brain injury location and type were found on any of the objective tasks. The results suggest that the overall design and interaction with the serious game was suitable for all types of brain injury patients in the sample.

Summarizing, in this study we have established what design choices should be made in order to enhance the usability of a serious game designed to train navigation strategies. From this first study, we can conclude that mouse controlled movement in 3D environments is more accessible than keyboard controlled movement. Video-based instructions are strongly preferred over text-based instructions, but not more effective in transferring knowledge. Feedback timing did not affect performance and motivation in the current training games. Based on the scores and usability questionnaires, the results suggest that usability of the serious game is adequate for the target patient population after the implementation of the appropriate features as determined in this study.

## Author Contributions

MvdK, JV-M, and IvdH developed the theoretical framework and conceived of the presented idea. JV-M contributed to organizing the experiments. MvdK carried out the experiment. MvdK wrote the manuscript with support from AE, JV-M, and IvdH. AE, JV-M, and IvdH provided critical feedback on the drafted manuscript. AE, JV-M, and IvdH supervised the project.

## Conflict of Interest Statement

The authors declare that the research was conducted in the absence of any commercial or financial relationships that could be construed as a potential conflict of interest.
