# Supplementary material for: A Usability Study of a Serious Game in Cognitive Rehabilitation: A Compensatory Navigation Training in Acquired Brain Injury Patients
Source: Front Psychol. 2018 Jun 5;9:846. doi: 10.3389/fpsyg.2018.00846 (PMC5996119; doi:10.3389/fpsyg.2018.00846)
Supplement: Supplementary file 1 [file Data_Sheet_1.pdf]

Supplementary Material

A usability study of a serious game in cognitive rehabilitation: a compensatory navigation training in acquired brain injury patients

Milan N. A. van der Kuil, Johanna M. A. Visser-Meily, Andrea W. M. Evers, Ineke J. M. van der Ham

Correspondence: Milan N. A. van der Kuil: m.n.a.van.der.kuil@fsw.leidenuniv.nl

Supplementary Table 1

Question/Statement	Response**<br>mean (SD)
Based on the past year … (ranging from yearly to daily)	
How often do you use a computer	4.43 (1.10)
How often do you play video games	1.9 (1.35)
Hof often do you use the arrow keys when playing videogames	1.57 (1.14)
How often do you use a joystick or controller when playing videogames	1.63 (1.13)
Please answer:	
I know how to start a computer	4.83 (0.75)
I know how use a computer mouse	4.83 (0.59)
I know how to use text editing software	4.53 (1.25)
I know how to use the internet to find things	4.87 (0.73)
I feel stressed when I use the computer*	4 (1.46)

Supplementary Table 1. Computer experience questionnaire. *Data shown on a reversed scale. **Ratings on a Likert scale with 1 corresponding to "completely disagree" and 5 corresponding to "completely agree". Standard Deviations appear in parentheses next to means.

Supplementary Table 2

Nr.	Question
	Sense of direction game
1	In this task I train my memory of landmarks (false)
2	I can confirm my pointing direction by pressing "Enter" key (false)
3	Whenever I have to point towards the stone a green arrow will appear (true)
4	The stone is located in the starting room (true)
5	If I point directly towards the stone I earn 1 coin (false)
6	I have to remember where the ending location is (true)
7	When the wall become transparent I can reorient myself (true)
8	When I use this strategy in the real world I do not have to remember landmarks (true)
9	I can imagine my sense of direction as a compass pointing to a certain location (true)
10	It is important I form a mental map of the environment (false)
11	I have to remember the layout of the corridors (false)
12	When I have to point to the stone I can always walk back (false)

Supplementary Table 2 Questions following instruction text/video for game A.

Supplementary Table 3

Nr.	Question
	Map use game
1	In this task I train my sense of direction (false)
2	In this task I learn how to interact with maps (true)
3	I have to walk towards the room containing the minotaurs (false)
4	I must try to visit as few rooms as possible (true)
5	I lose a coin whenever I enter a dead-end corridor (false)
6	The landmarks in the maze are shown on the minimap (true)
7	Planning of the route is important in the last round (true)
8	I have to remember how many minotaurs are in the maze (false)
9	I can use the dead-ends to determine my location on the map (true)
10	If I master this exercise, I will become better at using google maps to navigate in a city (true)
11	The tasks will be easier when there a more rooms in the maze (false)
12	The best strategy is to always turn left (false)

Supplementary Table 3 Questions following instruction text/video for game B.

Supplementary Table 4

Round	Cumulative Feedback mean (SD)	Delayed Feedback mean (SD)
All	3.48 (1.63)	3.95 (1.75)
1	1.00 (0.84)	1.43 (0.93)
2	1.48 (0.87)	1.67 (0.66)
3	1.00 (0.95)	0.86 (0.96)

Supplementary Table 4 Objective performance in the feedback timing task. Average number of coins are shown for each round. Two coins could be collected per round.
